# Microscopic Modelling Circadian and Bursty Pattern of Human Activities

**DOI:** 10.1371/journal.pone.0058292

**Published:** 2013-03-11

**Authors:** Jinhong Kim, Deokjae Lee, Byungnam Kahng

**Affiliations:** 1 Complex System Analysis Team, NHN Corp., Seongnam City, Gyeonggi-do, Korea; 2 Department of Physics and Astronomy, Seoul National University, Seoul, Korea; Umeå University, Sweden

## Abstract

Recent studies for a wide range of human activities such as email communication, Web browsing, and library visiting, have revealed the bursty nature of human activities. The distribution of inter-event times (IETs) between two consecutive human activities exhibits a heavy-tailed decay behavior and the oscillating pattern with a one-day period, reflective of the circadian pattern of human life. Even though a priority-based queueing model was successful as a basic model for understanding the heavy-tailed behavior, it ignored important ingredients, such as the diversity of individual activities and the circadian pattern of human life. Here, we collect a large scale of dataset which contains individuals’ time stamps when articles are posted on blog posts, and based on which we construct a theoretical model which can take into account of both ignored ingredients. Once we identify active and inactive time intervals of individuals and remove the inactive time interval, thereby constructing an *ad hoc* continuous time domain. Therein, the priority-based queueing model is applied by adjusting the arrival and the execution rates of tasks by comparing them with the activity data of individuals. Then, the obtained results are transferred back to the real-time domain, which produces the oscillating and heavy-tailed IET distribution. This microscopic model enables us to develop theoretical understanding towards more empirical results.

## Introduction

In the information age, a large scale of databases containing information on human activities on the Web are easily accessible. Understanding the emerging patterns from those datasets is a new interdisciplinary research subject [1], [2]. Since individuals behave through complex and sometimes random decision-making processes, one may wonder whether it is indeed possible to predict human behaviors quantitatively. However, it was recently revealed that digital records left at the media behind one’s activities make it possible to predict human activities up to 93% [3]. Accordingly, it has become an attractive subject to investigate emerging patterns from such large-scale data bases. Power-law or heavy-tailed behavior in the distribution of inter-event times (IET) between two consecutive human activities is one example of such emerging patterns. This example can be seen in various systems such as email [4]–[9] or surface mail communications [10], Web browsing [7], [11], library loans [7], financial trades [7], [12], on-line movie watching [13], file downloads [14]–[16], printing requests [17], and various actions on the Web [18]. This power-law behavior indicates that human activities proceed in a bursty manner during a short time interval, which is separated from other such intervals by long intermittent periods [19], [20].

Several theoretical models have been proposed to explain such heavy-tailed behaviors in the IET distribution. One interesting model is the priority-based queueing model [4], [7], [21], [22], in which the human activity of uploading articles is regarded as task executions in a queue, where tasks are performed based on the order of priorities assigned to each task. The use of this priority-based model leads to a power-law or heavy-tailed behavior in the waiting time distribution of tasks in the queue [9], [13], [18], [23]–[25]. The waiting time distribution was interpreted as the IET of human activities. However, the priority-based queueing model ignores important ingredients, such as the circadian pattern of human life [26] and the diversity of individual activities. Indeed, the empirical data recently collected exhibit an oscillating pattern with a one-day period in the IET distribution [6], [13], [18], [27], which cannot be produced in the queueing model. Moreover, the decay behavior of the IET distribution in the long-time regime depends on the activities of individuals. Here, the activity of an individual is defined as the average number of posted articles in unit time. In this paper, we obtained a large-scale dataset containing high-resolution data, and found a new pattern in the IET distribution that exhibits a power-law behavior when the IET is smaller than one day, where the exponent is insensitive to the activities of individuals. However, when the IET is longer than one day, the IET distribution exhibits a heavy-tailed behavior, in which the tail part depends on the activities of individuals. These empirical results are reproduced by developing a theoretical model below.

## Methods

We analyze a large scale of dataset from the largest portal site in Korea, NAVER (http://naver.com) during more than five years. The dataset consists of individuals’ time stamps when articles were posted on blog posts, which were recorded in the unit of seconds. There are 520,771,167 postings contributed by 9,878,904 distinct bloggers. Among them, we only select the data that were written by bloggers that had authored more than 100 articles and worked for more than one month. This selection aims to exclude those bloggers who had posted suspicious spam content. After this data filtering, the number of remaining articles is 379,627,193, contributed by 908,409 users.

From this dataset, we obtained the following empirical results: (i) The IET distribution decays following a power law with the exponent 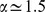 in a time regime shorter than one day. (ii) The IET distribution exhibits a heavy-tailed decay behavior in the long-time regime, which is nonuniversal depending on individual activities. (iii) An oscillating pattern appears with a period of one day; this pattern persists over the entire long-time regime. However, the amplitude of the oscillation pattern decreases with time. Details regarding these results are presented below.

**Figure 1:**
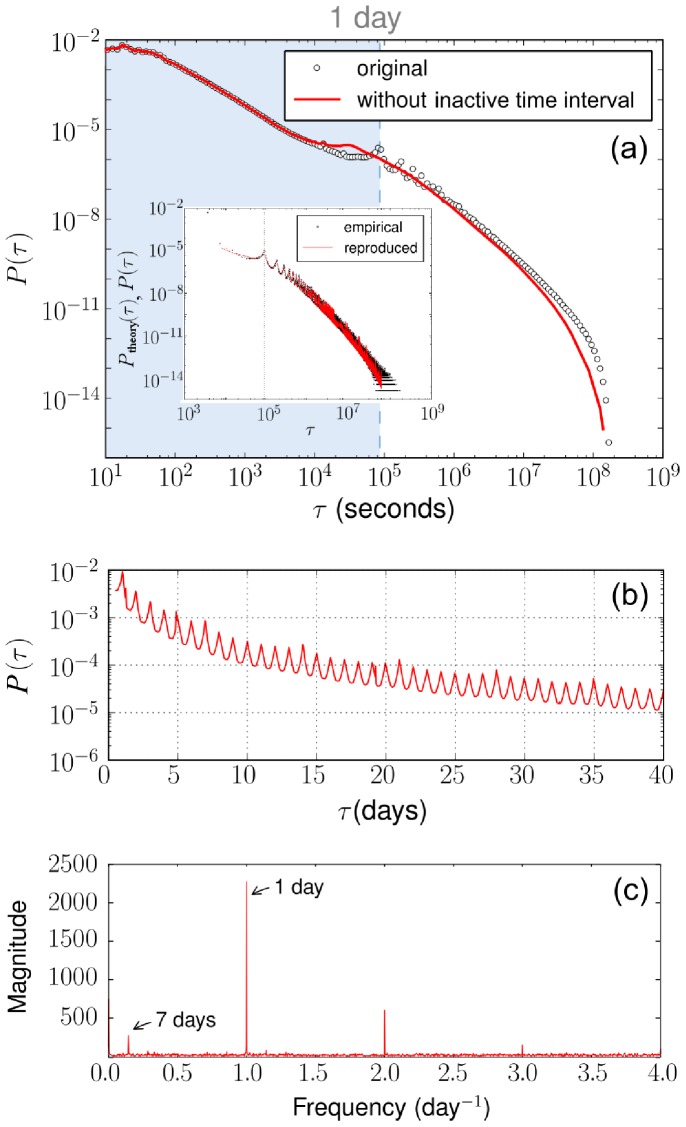
Empirical IET distribution. (a) Plot of the IET distribution 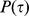 based on the empirical data (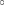). The IET distribution 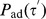 after the removal of the inactive time interval is also shown (solid curve). Inset: Comparison of the IET distribution obtained from the empirical data (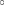) with that from the theory 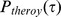 (solid curve). (b) Enlarged representation of the IET distribution 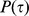, in which clear periodic peaks are observed. (c) The Fourier transform of the IET distribution. Peaks are located at frequencies 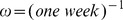 and 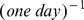. Other peaks at multiples of 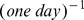 are redundant.

We measured the IETs defined as the interval between two consecutive time stamps for each user. Then the distribution 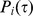 of the IETs of user 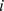 is obtained as 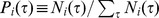, where 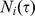 is the number of events having an IET of 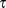. The total number of articles, 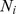, written by user 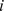 is given as 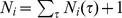. To determine the collective behavior of all the users, we calculate
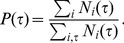
(1)

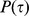
 is shown in Fig. 1(a). When 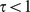 day, 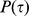 behaves as 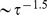. When 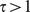 day, 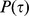 follows a skew distribution. Interestingly, there exists an oscillating pattern in 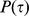, which can be seen more clearly in the finer scale shown in Fig. 1(b). Moreover, peak heights periodically change with a period of one week [6], [13], [18], [27]. To check the periodicity of the oscillating pattern, we perform a Fourier transformation, 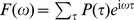. Figure 1(c) shows that there indeed exist two distinct meaningful peaks in 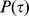 at the frequencies corresponding to one day and one week, respectively. Other peaks correspond to multiples of one day. We study how such an oscillating pattern can be reproduced within the framework of the priority-based model later.

**Figure 2:**
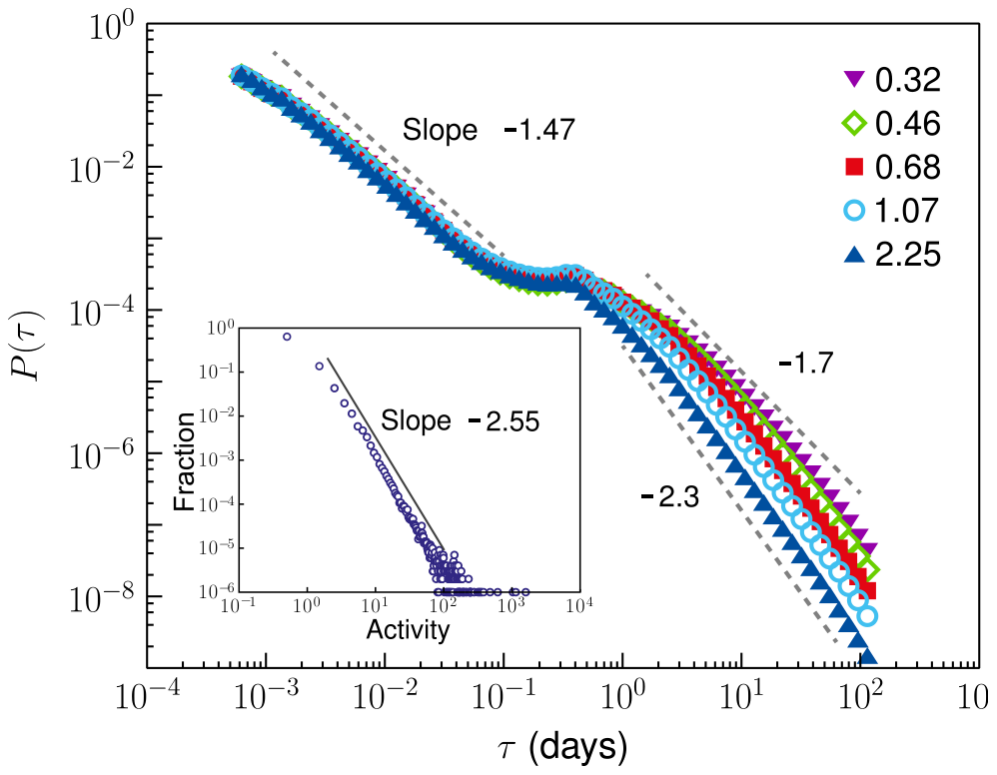
Dependence of the IET distributions of individual users on their activities. Inset: Population as a function of activity, showing decay as a power law with exponent 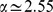.

Next, we examine the dependence of the IET distribution on the activity of individuals. The activity 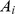 of user 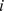 is the number of articles written per unit time interval. Thus, when user 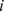 writes 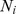 articles during the time interval 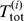 [13], [18], where 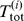 is the time interval between the first and the last time stamp of user 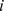, the activity of user 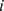 is given as 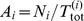. To determine the heterogeneity of individual activities, we measured the distribution of individual activities as shown in the inset of Fig. 2. Indeed, the distribution decays, following a power law with the exponent 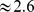, indicating that individual activities are considerably heterogeneous. Thus, it is worth investigating how the heterogeneity of activities affects the IET distribution [28], [29]. In Fig. 2, we can see that as one’s activity level becomes higher, the IET distribution decays faster in the long time regime. This behavior is rather natural in the sense that a user with higher activity has a shorter mean IET. Accordingly, it would be interesting to introduce a new model to illustrate this activity-dependent behavior, and such a model is presented later.

## Results and Discussion

### Modelling Oscillating Pattern

**Figure 3:**
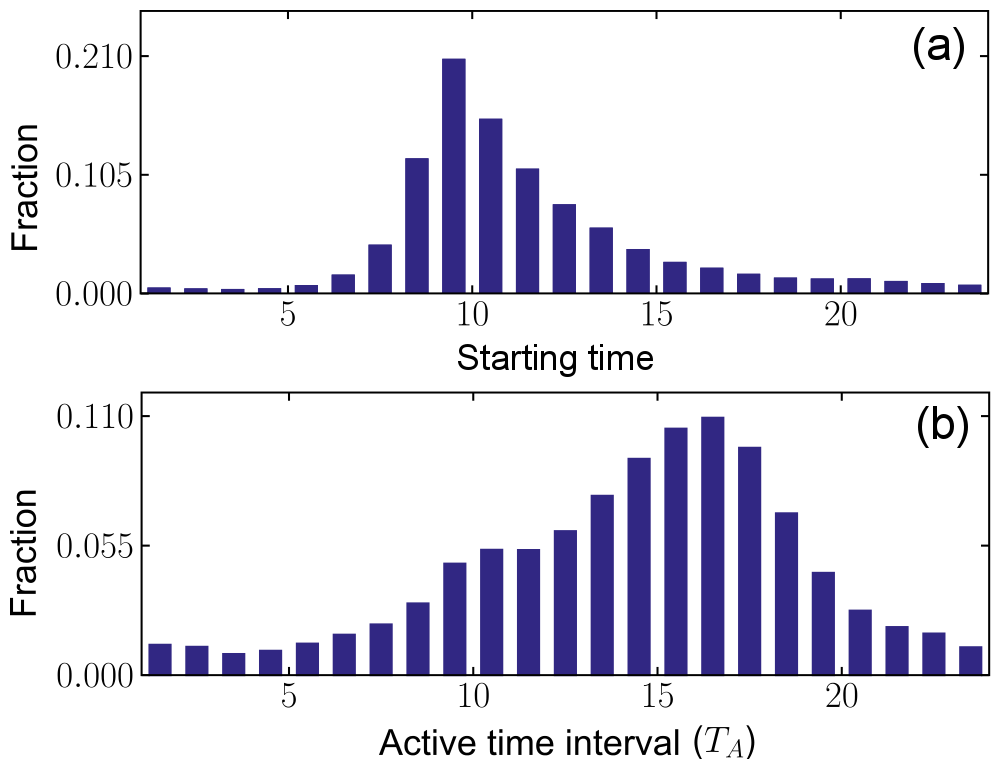
Distribution of active time intervals. (a) Distribution of the starting time of the active time interval. A peak is located between 9 and 10 am. (b) Distribution of active time intervals. The model is located at 16 h.

In previous studies, the heavy-tailed behavior of the IET distribution was investigated by using the priority-based queueing model. In this approach, time was considered as continuous without any intermission. However, humans do not work continuously, and hence, intermission, for example, those that account for sleeping, must be considered. Moreover, the pattern of daily life during weekdays is almost regular, but it differs from that during weekends. Thus, it is natural to assume that each person can have a regular time interval during which the person is away from on-line world. This time interval is called the inactive time interval, and the remaining time of a day is called the active time interval. Moreover, the duration and starting time of the active time interval depend on the individual (see Fig. 3).

**Figure 4:**
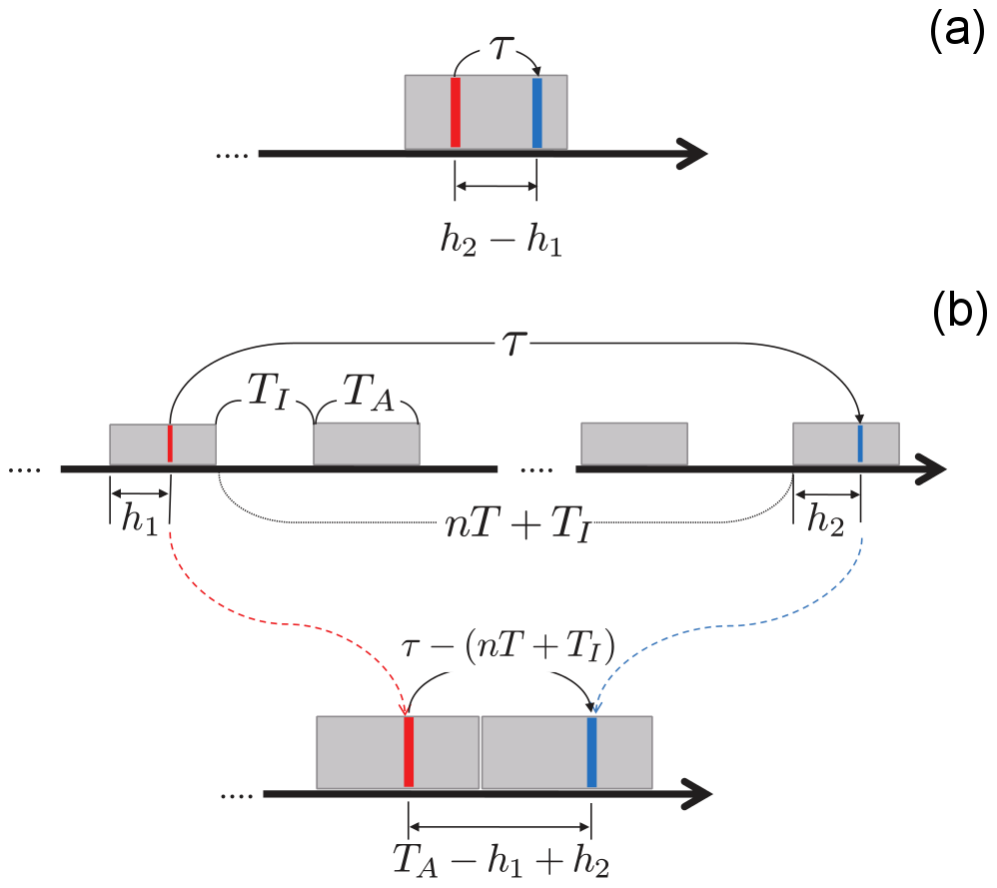
Schematic illustration of the model with circadian periodicity. It is assumed that an individual essentially lives a well-regulated daily life consisting of active and inactive time intervals. To reproduce the oscillating behavior of IET distribution within the framework of the queueing model, we construct an *ad hoc* time domain in which separated active time intervals are connected by removing inactive time intervals between them. See text for details.



We suppose the situation that two events occur in the active period of one day (see Fig. 4a) at times 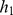 and 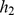, where 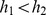 and 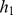 and 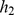 belong to the same active time interval. Then, the inter-event time is defined as 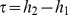. More generally, when two events are executed in different active intervals separated by 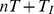, where 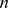 is an integer 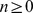 (see Fig. 4b), we can obtain the following relation,
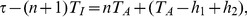
(2)where 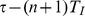 is the IET after removing the inactive time intervals. This quantity is defined as the IET in the *ad hoc* time domain, and is denoted as 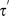. Then the *ad hoc* time domain is continuous. We find that any inter-event time 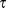 belongs to one of the two sets of intervals 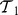 and 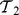, defined as
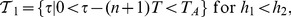
(3)and

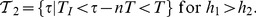
(4)The fraction of each category is given as
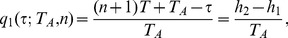
(5)and
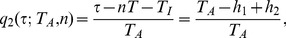
(6)respectively.

Let 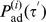 be the IET distribution of user 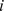 in the *ad hoc* time domain, and let 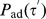 be the collective one from individuals, defined as
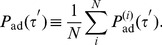
(7)


Here, 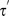 is the IET defined in the *ad hoc* time domain, which is related to 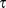 in the original time domain as 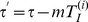, where 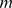 is the largest non-negative integer satisfying 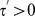, which implies that there exist 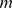 inactive time intervals during 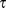. 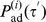 is obtained from the queueing model [30], which is discussed later. Collecting all individuals’ 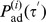, i.e., using the formula (7), we obtain 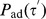, which exhibits a heavy-tailed distribution shown in Fig. 1.

**Figure 5:**
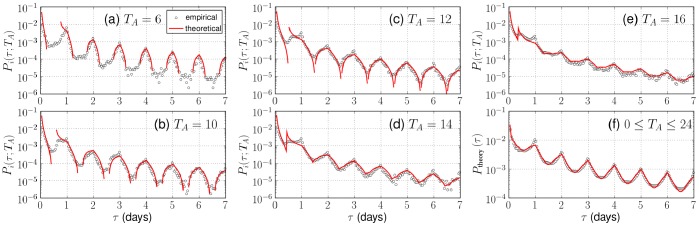
Comparison of empirical (open circles) and theoretical (solid lines) inter-event time distributions with the circadian active-inactive pattern for different 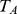. Empirical distributions 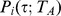 are obtained by aggregating the top 100 users who have a clear periodicity with an active time interval 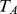, and the distributions suitably show the change in the peak height and width. The weighted average of 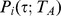 is also displayed in (f), and we can observe the characteristic peaks.

**Figure 6:**
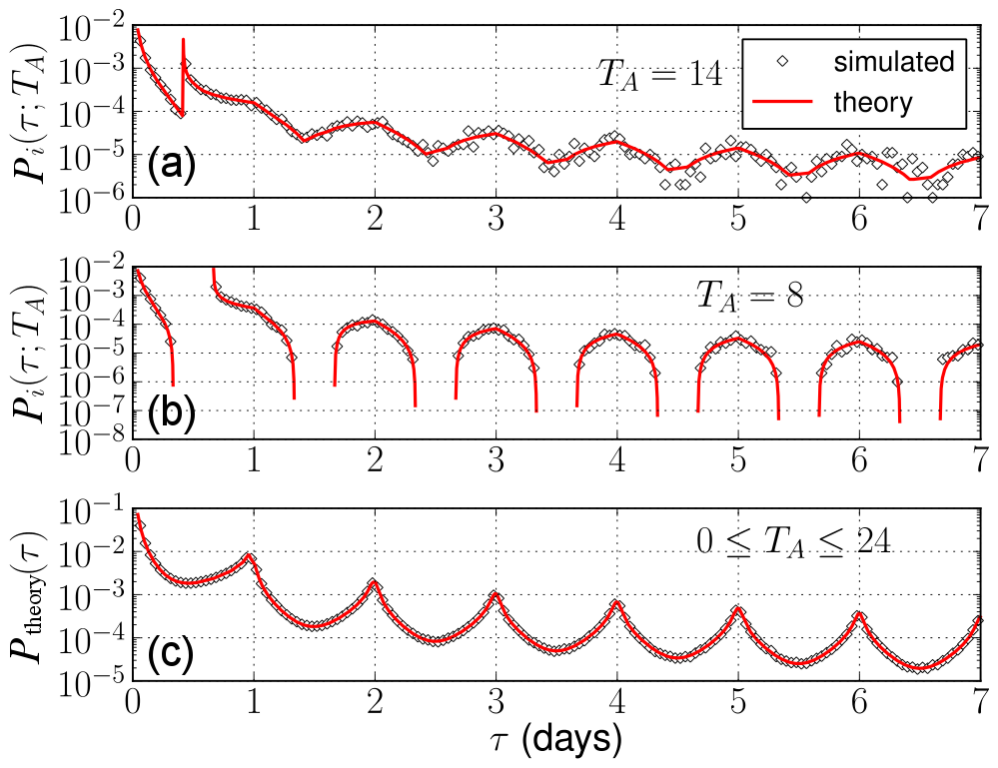
Comparison between simulated and theoretical IET distributions with the circadian pattern. To calculate 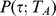 in Eq.(8), we assume that 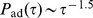. We consider the two cases (a) 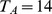 and (b) 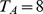, as examples. (c) The distribution in Eq.(10) collected over the flat distribution of 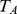. The resulting theoretical IET distribution is consistent with the one obtained from the simulated data.

We consider how to reproduce the oscillating behavior. For this purpose, we assume that an IET distribution is given, for example, the previously obtained 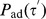 from the empirical data, or 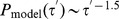 from the queueing model [30]. Then, we can obtain the IET distribution of user 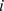 with the active time interval 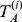 as follows:
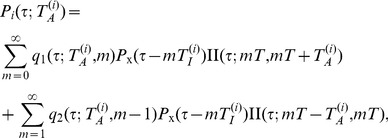
(8)where 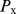 represents either 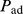 or 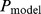. 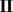 is a rectangle function defined as
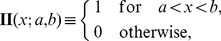
(9)which represents the intervals defined in 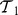 and 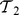. Next, we obtain the average 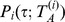 over all users and obtain
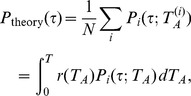
(10)where 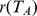 is the fraction of users whose active time interval is 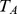. The distribution of 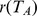 exhibits a peak at 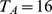 h as shown in Fig. 3(b). By plugging the empirical distribution 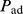 into 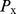 in Eq. (8), we successfully reproduce the oscillating pattern of the IET distribution 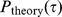 in the inset of Fig. 1(a) and Fig. 5. When 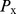 is replaced by the theoretical formula 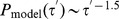 [30], the obtained result for 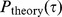 is consistent with the simulated one, as shown in Fig. 6. It is noteworthy that the functional form of 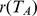 does not play an important role in determining the oscillating behavior of the IET distribution. For example, even for the flat distribution of 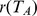, the oscillating pattern of 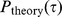 can be obtained.

### Modelling Activity Dependence

As discussed in the previous section, we have shown that the activities of individuals are heterogeneous and that their distribution follows a power law: 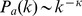 with 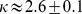 as shown in the inset of Fig. 2. That is, a few people post many articles and many others post only a few articles in a given interval. Moreover, individuals have their own active time intervals. Thus, it would be interesting to study how such heterogeneities affect the IET distribution. We categorize users into groups according to their activities, and we measure the IET distributions of each group as shown in Fig. 2. It is interesting to notice that the IET distribution appears to be independent of activities in the short-time regime within one day, but it depends on activities in the long-time regime.

In the priority-based queueing model introduced in Ref. [30], packets arrive at a queue with the rate 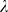 and are executed with the rate 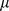, where the rates 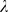 and 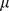 are regarded as constants, independent of time and individuals. Here, however, since the activity and the period of the active time interval are different, we assign user index 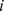 to the rates as 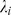 and 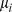, and those quantities are assumed to depend on time. We consider 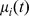 as proportional to the frequency of blog postings at time 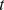 by user 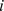. Next, we use the following relation between the execution rate 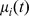 and the activity 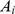,
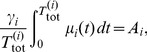
(11)where 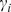 is a proportionality constant. For the arriving rate 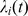, since we do not have any information of when a new task is arriving, we assume 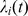 to be the same as 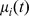.

Based on this idea, for each user 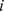, we perform numerical simulations as follows:

- We numerically generate both arrival and execution time sequences 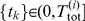 through the Poisson process with the rates 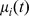 and 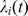 [31].
- Subsequent these time sequences, we input a task into the queue when it is not full of 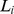 tasks, where the queue size *L*i is determined at a later stage. Upon arrival, the task is given a priority 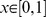. At the same time, a task with the highest priority is executed and removed from the queue. The waiting time of the task is also recorded.
- We repeat this procedure until *N_i_* waiting times are obtained. *N_i_* is regarded as the number of blog posts uploaded by user 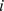.

In this model, the activity is determined to be 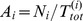, whereas the queue size 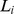 and the proportionality constant 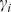 remain to be determined.

**Figure 7:**
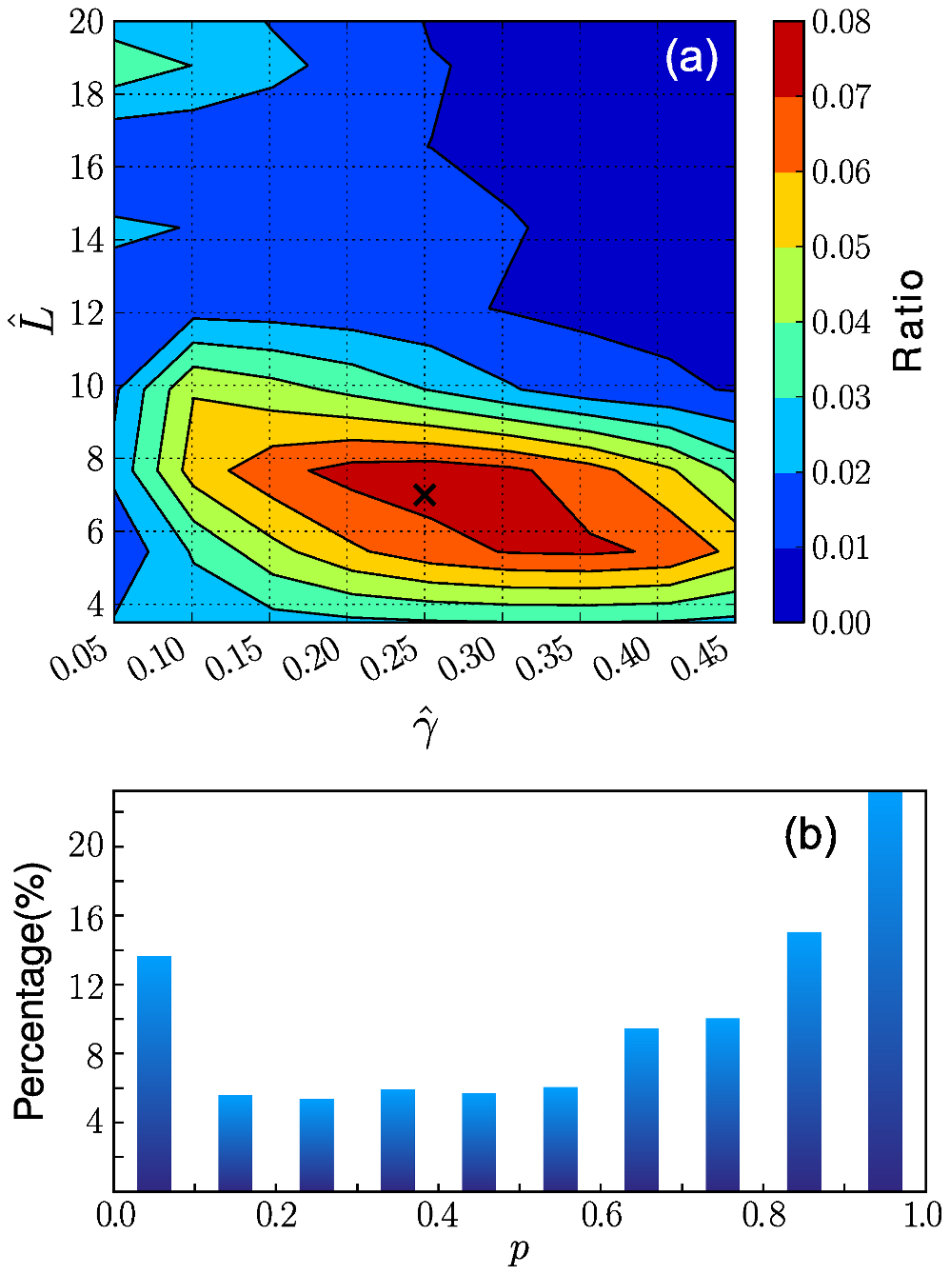
Modelling activity dependence. (a) Distribution of the best-estimate parameters 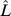 and 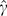 of individuals. Contour lines are obtained by interpolation between each nearest point. The most dense point is described by 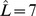 and 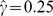, and a large portion of cases settle around the peak point. (b) A fraction of the 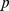 values in the KS test between synthetic and empirical probability distribution functions. Over 86% of cases have 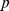 values that are larger than 0.1, and hence, the null hypothesis cannot be rejected for those cases.

**Figure 8:**
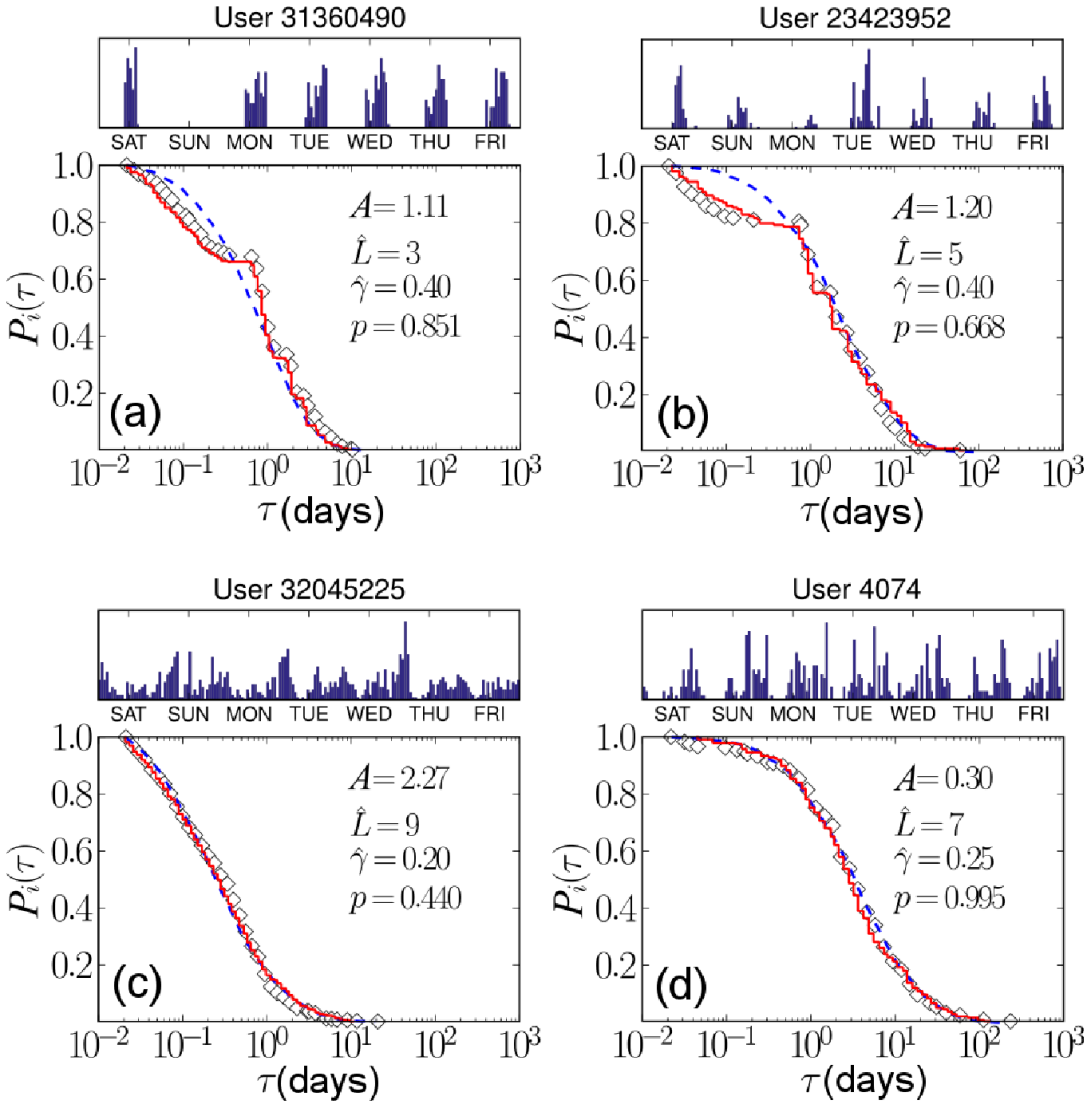
Comparison between empirically observed individual inter-event time probability distributions (open diamonds) and model predictions that are fit to the data. Model predictions are calculated by two methods by using 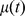 and 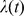 (red solid lines), and by using time-averaged rates of 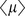 and 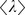 (blue dotted lines). The histograms in the upper panel of each plot represent the relative ratio of blog posts written during a certain hour of the day. In cases with clear periodicity (a) and (b), red solid and blue dotted lines show apparent differences. Otherwise (c) and (d), they exhibit very similar patterns, and the periodicity assumption seems to be irrelevant to them. On the other hand, we only consider data points on scales larger than 30 min, because the resolution of 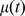 and 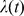 is 1 h.

To determine 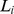 and 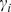, i.e., to generate a synthetic probability distribution function fit to the empirical data, we use the Kolmogorov-Smirnov (KS) statistical test [32]. We obtain a set of 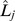 and 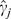 for each user 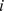 by minimizing the KS statistic between the empirical data and simulated data. They are distributed as shown in Fig. 7. The closeness between the empirical data and the simulated data is tested (see Fig. 8): the obtained 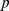 value is shown in the legend. It is known that if the 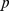-value is higher than a preassigned value (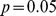), then one can accept the null hypothesis that the probability distribution functions are identical. As we can see in the 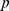-value histogram of Fig. 7(b), most cases show good agreement between synthetic and empirical data with high 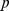 values: The fraction of users is 23.2% for 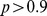, and 86.3% for 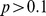. Thus, it can be said that our theoretical result reasonably reproduces the empirical pattern.

Moreover, we simulate the queuing process by using the average rates of 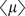 and 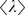 instead of the time-dependent form of 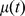 and 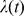 for each user. In most cases, there is only a slight difference between the two simulated results with different types of parameters as shown in Fig. 8. However, there are apparent different cases for the two results; these occur when periodic time intervals appear in the activity of writing blog posts. In this case, the time-dependent forms 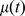 and 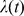 are better for fitting to the empirical data.

### Conclusions

In this work, we have studied the inter-event time statistics of human dynamics based on a large scale of on-line records of blog writings at a Korean portal site. We observed that the IET distributions of each user exhibit a universal pattern in the short-time regime, but they exhibit different decay patterns in the long-time regime, which depends on the activities of individual users. Moreover, we observed a clear periodic pattern with a period of one day, which reflects the circadian pattern of human behavior. We explained these patterns within the framework of the queueing model. First, we identified active and inactive time intervals of individual behaviors and then removed inactive time interval and constructed an *ad-hoc* time domain. Next, we applied the priority-based queueing model in the *ad-hoc* time domain by adjusting the arrival and execution rates of tasks to the empirical data. Following this, we returned to the real time domain and found our theoretical results to be in agreement with the empirical results including the positions of circadian peaks [6], [13], [18], [27]. The microscopic studies performed in this paper enable us to understand these empirical results from a theoretical perspective.
